# Feasibility of the modified inverted L-shaped approach for posterolateral tibial plateau fracture: A retrospective study

**DOI:** 10.1097/MD.0000000000031057

**Published:** 2022-10-07

**Authors:** Xiaohai Yang, Mingming Pan, Hanliang He, Weimin Jiang

**Affiliations:** a Department of Orthopedics, Suzhou Dushu Lake Hospital, Jiangsu, PR China; b Department of Orthopedics, the First Affiliated Hospital of Soochow University, PR China.

**Keywords:** clinical efficacy, posterolateral inverted L-shaped approach, posterolateral tibial plateau fracture

## Abstract

Approaches for treating posterolateral tibial plateau fractures vary among surgeons, and the inverted L-shaped approach is a known option. This article aims to introduce a new modified posterolateral inverted L-shaped approach for isolated posterolateral tibial plateau fractures and study its feasibility. Medical records of patients with posterolateral tibial plateau fractures were reviewed. Plain radiographs were obtained during the follow-up period, and the hospital for special surgery (HSS) score was used to assess the function of the injured limb. Perioperative complications were recorded and followed-up. In total, 32 patients with posterolateral tibial plateau fractures were treated using a modified posterolateral approach. The mean age of the patients was 44 ± 11 years (28–64 years). All patients successfully underwent surgery and were followed-up for a mean duration of 13 ± 2 months (10–16 months). On plain radiographs, fracture lines were fuzzy 3 months after surgery and disappeared 12 months after surgery. No perioperative complications occurred during the follow-up period. The HSS score was evaluated 12 months after surgery, and the mean score was 91 ± 5 points (81–97 points), including 25 excellent cases and 7 good cases. The modified posterolateral inverted L-shaped approach has the advantages of small soft tissue dissection, fracture reduction under direct vision, easy internal fixation, and a lower risk of neurovascular injury. This approach is feasible for the treatment of isolated posterolateral tibial plateau fractures, and further high-quality randomized control trials are required to confirm its clinical efficacy.

## 1. Introduction

Posterolateral tibial plateau fracture is relatively rare in clinical practice and accounts for approximately 7% of total tibial plateau fractures.^[1]^ The mechanism of this type of fracture is valgus, in which axial force is placed on a flexed or semi-flexed knee joint. The sheath force of the lateral femoral condyle is directly applied to the posterolateral tibial plateau surface and results in collapse or splitting fracture, although the latter is uncommon owing to the support of the fibular head.^[2]^ Posterolateral stability of the knee joint is impaired in this type of fracture, and further joint disability and abnormal activity may persist. The local stress increases by 75% when the joint surface step reaches 3 mm. As the number of steps increases, cartilage wear increases accordingly. As a result, joint degeneration is aggravated and knee joint function is affected.^[3]^

Due to the poor outcomes of posterolateral fractures, conservative treatment is not recommended. Several approaches have been introduced for the management of isolated posterolateral tibial plateau fractures, such as the posterior approach, posterolateral approach with or without fibular osteotomy, and modified anterolateral approach.^[4]^ These approaches have some disadvantages such as large trauma, bleeding, risk of neurovascular damage, and difficulty in internal fixation.^[4]^ In recent years, the inverted L-shaped approach has emerged as an alternative treatment for isolated posterolateral fractures. In this article, we introduce our modified inverted L-shaped approach, share its clinical outcomes, and explore its feasibility.

## 2. Methods

### 2.1. General information

This retrospective study was approved by the ethics committee of the local hospital. Patients with isolated posterolateral tibial plateau fractures between March 2011 and September 2019 were treated using a modified inverted L-shaped approach. The inclusion and exclusion criteria were as follows: fresh and closed tibial plateau fracture; no pathological fracture caused by malignancy or metastasis; no symptoms of neurovascular injury; and no injury to other organs and good medical condition.

### 2.2. Surgical methods

**Figure 1.**
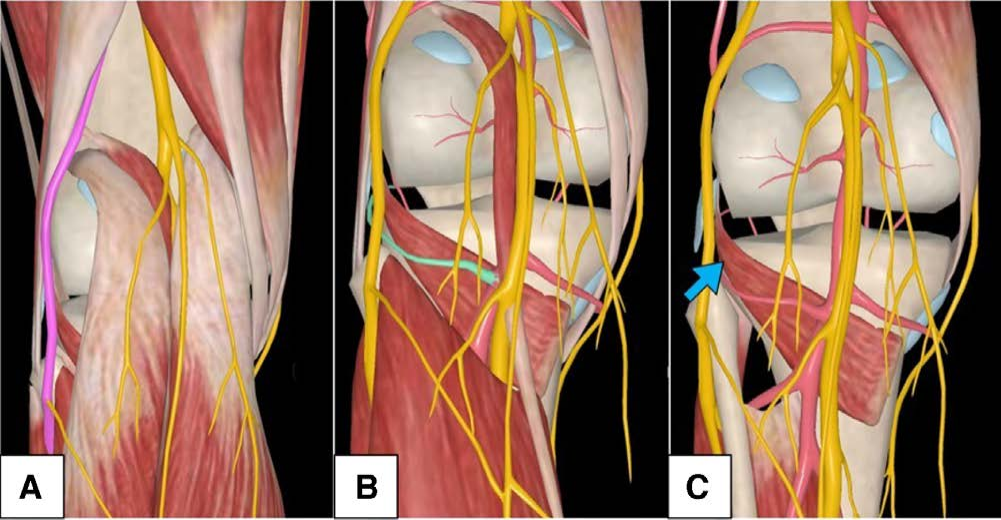
Schematic illustration of anatomic structures of inverted L-shaped approach. (a) Common fibular nerve (pink line) was exposed and protected, and the lateral head of gastrocnemius muscle was stretched inward. (b) Lateral inferior genicular artery (green line) was ligated. (c) Soleus muscle was bluntly dissected and the popliteus tendon was stretched inward and upward (blue arrow).

After general anesthesia, patients were placed prone or lateral on the operation table, with a fractured limb on the upper side. The incision was made from the middle part of the popliteal dermatoglyphics and vertically turned down at the lateral end of the popliteal dermatoglyphics. The incision was then extended along the outside of the lateral head of the gastrocnemius muscle and ended at a point 6 cm from the fibular head. Subcutaneous tissues were cut and dissected, and the common fibular nerve was carefully exposed and protected. The lateral head of the gastrocnemius muscle was stretched inward, and the lateral inferior genicular artery was ligated. The soleus muscle was bluntly dissected from the posterolateral end of the proximal tibia, and the popliteus tendon was stretched inward and upward. The posterolateral complex was cut, and the fracture was exposed (Fig. 1). The fracture was restored and temporarily fixed using K-wires, and a 3.5-mm T-shaped buttress plate (Synthes, Switzerland) was used to fix the fracture. The subcutaneous fascia and skin were sutured sequentially after the operation area was washed with diluted iodophor and normal saline. Finally, the incision was covered with sterile dressing and bandaging.

### 2.3. Postoperative management and follow-up

Passive exercise was conducted with a continuous passive motion device 2 days after surgery, and the range of motion started at 30°, which was supposed to increase to at least 90º in 2 weeks. Active motion of the knee and ankle joints was necessary. Three months after surgery, patients were encouraged to walk with partial body weight. Plain radiographs were obtained at 2 days, 1 month, 3 months, six months, and 12 months after surgery to evaluate the healing progress of the fracture. The hospital for special surgery (HSS) score was used to assess the function of the injured knee joint and included six parts: pain (30 points), function (22 points), range of motion (18 points), muscle strength (10 points), knee flexion deformity (10 points), and stability (10 points).^[5]^

## 3. Results

**Figure 2.**
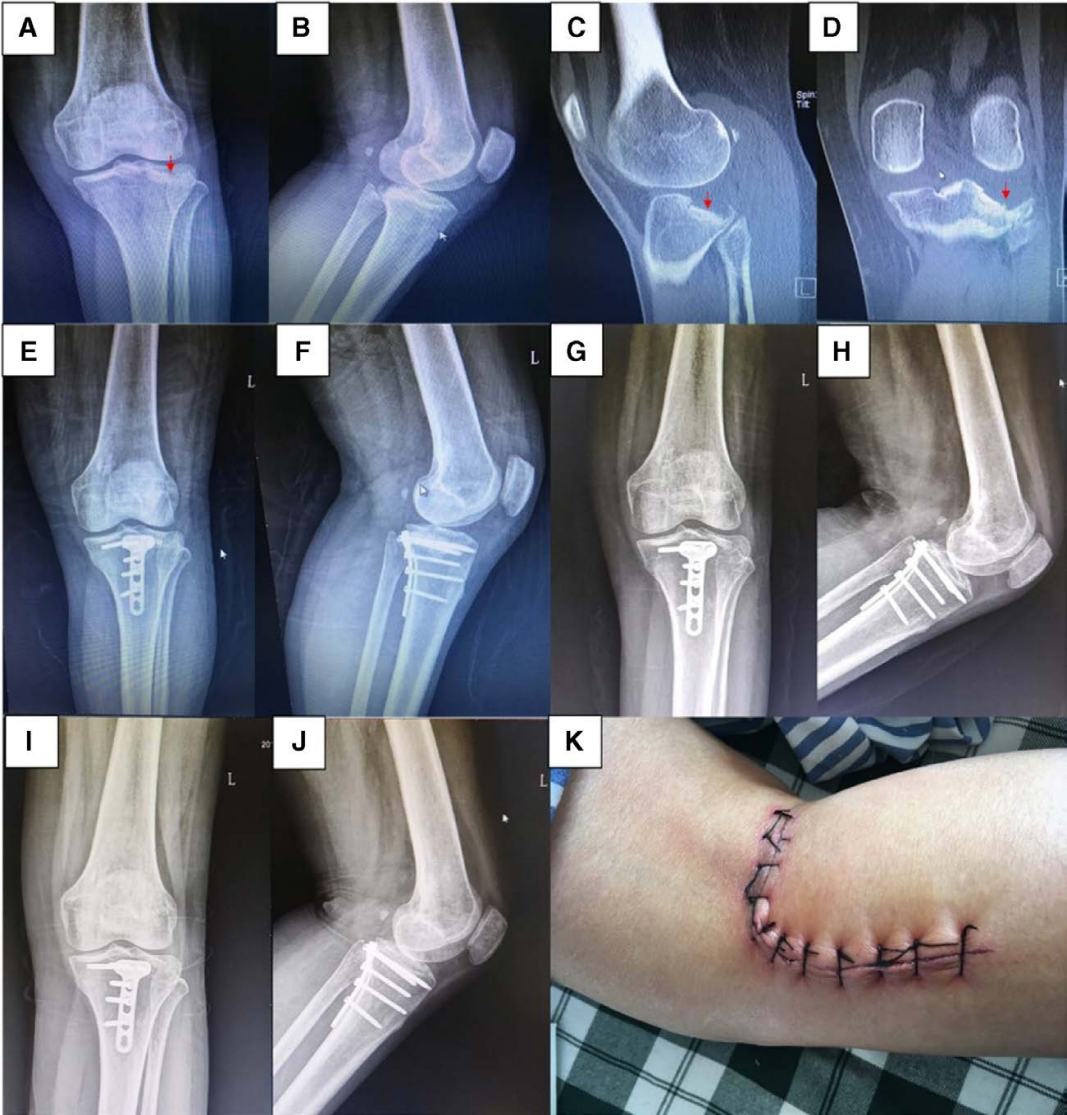
A 55-year-old female suffered from posterolateral tibial plateau fracture caused by traffic accident. X-ray and CT scan (a–d) revealed a Schatzker type III fracture with subsidence of lateral surface (red arrow). Postoperative radiograph 2 days (e, f), 3 months (g, h) and 12 months (i, j) after surgery showed the restoration of lateral joint surface and stable internal fixation. No obvious recollapse occurred during follow-up period. Skin tension was eased by the use of popliteal dermatoglyphics (k).

A total of 32 patients (18 men and 14 women) with posterolateral tibial plateau fractures were treated, and all were followed-up. Among them, 16 patients suffered from traffic accidents, 13 patients fell over themselves, and the other 3 patients were hurt by falling from height. The mean follow-up duration was 13 ± 2 months (10–16 months) (Table 1). On plain radiographs, fracture lines were fuzzy 3 months after surgery and disappeared 12 months after surgery. The mean HSS score was 91 ± 5 points (81–97 points) at the last follow-up period, with 25 patients reporting excellent outcomes and 7 reporting good outcomes. Additionally, 3 patients had internal fixation removed 1 year after surgery. The incisions healed well, and no perioperative complications such as infection, neurovascular injury, internal fixation failure, or joint surface re-collapse occurred (Fig. 2).

**Table 1:** Demographic data of patients.

Item	Data	
Total number (n)	32	
Age (yr)	44 (±11)	
Gender (Male/Female)	18 (56.3%)/14 (43.7%)	
Schatzker classification	II	8
	III	17
	V	5
	VI	2
Follow-up duration (mo)	13 (± 2)	
Complications	None	

## 4. Discussion

### 4.1. Feasibility and efficiency of modified posterolateral inverted l-shaped approach

Open reduction is important in the treatment of tibial plateau fractures. Singleton et al revealed that patients could achieve better range of motion, higher knee injury, and knee injury and osteoarthritis outcome score with less pain if joint surface subsidence was less than 2.5 mm after surgery.^[6]^ Parkkinen et al also reported that subsidence of >2 mm and varus of >4° were risk factors for postoperative knee arthritis.^[7]^ Both of these studies indicate that anatomical reduction is crucial to great clinical outcomes, and one of the key factors to achieve anatomical reduction is correct selection of the surgical approach. Recently, direct approaches to treat posterolateral plateau fractures have attracted great attention from surgeons.^[8,9]^ Tao et al first reported a posterolateral inverted L-shaped approach for the treatment of posterolateral plateau fractures in 2008.^[10]^ In their study, the mean recovery time of fractures was 12 weeks, and the mean HSS score reached 93 points (84–97 points), with no perioperative complications such as incision infection, skin necrosis, or fixation failure. Luo et al^[11]^ also carried out a cadaveric study on 16 knee specimens to explore the safety and efficacy of the posterolateral inverted L-shaped approach.

The results proved that this approach was capable of meeting the need for open reduction and internal fixation for posterolateral tibial plateau fractures, with great caution to avoid damaging the pretibial vessels. We modified Tao’s approach by starting from the middle part of the popliteal dermatoglyphics, instead of the medial part. The modified approach shortened the length of the incision, decreased the risk of neurovascular injury, and provided adequate space for surgery. The modified approach also ended 6 cm from the fibular head, instead of 8 cm from the tibial plateau. Heidari et al conducted a cadaveric study on 40 unpaired adult knee specimens, which showed that the distance from the interosseous membrane hiatus to the joint surface was 46.3 ± 9.0 mm (27–62 mm), and it was 35.7 ± 9.0 mm (17–50 mm) to the tip of the fibula head.^[12]^ We believe that the joint surface was not reliable as an anatomical landmark due to its subsidence and loss of primary height. However, the fibula head rarely suffered damage to the fracture and was easy to touch from the body surface, making it a more appropriate reference endpoint.

### 4.2. Advantages and disadvantages of the posterolateral inverted l-shaped approach

Compared with other approaches, the posterolateral inverted L-shaped approach has several advantages, including the following: instead of the anterolateral approach, the posterolateral approach can reduce and fix fractures under direct vision.^[13]^ The single posteromedial approach makes treatment of posterolateral fractures difficult without assistive anterolateral incision, and a cadaveric study implied that posteromedial could only expose 43.72% of the lateral joint surface, which was significantly increased to 81.41% by the use of posterolateral inverted-L approach.^[14]^ The S-shaped incision behind popliteal fossa has great risk of neurovascular injury and scar contracture, while a posterolateral inverted-L shaped approach includes a lesser wound and risk. The fibula osteotomy approach also results in considerable trauma as well as disability of lateral knee joint. Osteotomy of the fibula head also increases the risk of iatrogenic common peroneal nerve injury and nonunion.^[15,16]^ Our modified approach causes less damage to the lateral knee joint and can protect the common peroneal nerve by isolating and stretching it upward. With the use of popliteal dermatoglyphics, it is easy to stretch the lateral gastrocnemius muscle and other structures to the medial side using our modified approach. Popliteal dermatoglyphics are also helpful for easing skin tension. In contrast, the posterolateral straight and Carlson S-shaped approaches are longer than our modified approach in length and have a greater risk of knee dysfunction due to scar contracture. In the study of He et al, they addressed that both posterolateral and posteromedial fracture could be reduced and buttressed directly. Moreover, the articular surface could be achieved and supported through this approach.^[17]^

In summary, we believe that the modified posterolateral inverted L-shaped approach has several advantages and meets the requirements for treating isolated posterolateral tibial plateau fractures. However, this approach has several limitations. First, exposure of the surgical area is limited owing to the restriction of the anterior tibial artery and other vessels, which makes it difficult to expose the joint surface under direct vision. The exposure of the lateral plateau may be more difficult if the patient is heavy or exceptionally muscular. Second, improper traction during reduction has the risk of damaging the anterior tibial artery and other significant neurovascular structures in the popliteal space.^[18]^ Finally, the modified approach has a relatively limited surgical field to deal with complex fractures. When both the anterolateral and posterolateral columns are involved in a fracture, an additional anterolateral approach is necessary. The Frosch approach is also feasible for this type of fracture.^[15]^ If the fracture line is long downward or involves the posteromedial column, the approach should be lengthened or replaced with the Carlson approach.

### 4.3. Indications and complications

There are a few indications for our modified inverted-L shaped approach. Regarding our practice, we recommend patients with isolated posterolateral tibial plateau fracture. Moreover, He et al reported an inverted-L shaped approach applied to treat bicondylar tibial plateau fractures, an application which needs further study and confirmation.^[17]^ Regarding complications, the most severe known complication is injury of neurovascular bundle during exposure progress. Cutaneous necrosis is another high-risk complication due to the shape of the incision and the stretch or exposure of skin. This approach may sometimes result in the damage of the posterolateral complex, which is important to the stability of knee joint, during exposure progress.

### 4.4. Our surgical experiences

The angle of the incision should be rounded, instead of rectangular, to avoid skin necrosis. The common fibular nerve should be properly exposed and protected. The traction of muscles must be soft for the anterior tibial artery and other significant neurovascular structures in the popliteal space. Further, the popliteus tendon is not suggested to be cut off, whereas the popliteofibular ligament and part of the popliteus muscle may be cut and repaired later when the tendon is difficult to stretch. Appropriate shaping of the plate is essential to fit the anatomy of the posterolateral joint surface. Screws should be as long as possible to most easily pass through the anterior bone cortex and to increase the stability of internal fixation. When the proximal tibiofibular joint surface is involved in fracture, 45° oblique osteotomy of the fibular head is beneficial for the exposure and reduction of fractured fragments, as well as the lateral placement of the plate. The starting point of osteotomy is the posterior medial side of the attachment points of the lateral collateral ligament and the biceps femoris tendon. Under normal circumstances, removal of the internal fixation after recovery is not recommended. We attempted to remove the posterolateral plates in 3 patients and found it difficult to separate the soft tissue due to severe scar adhesion. Consequently, one patient experienced symptoms caused by stretching of the common fibula nerve, which was revealed spontaneously during the follow-up period. Another patient developed hematoma under incision 4 days after the secondary surgery and was also healed by pressure dressing.

## 5. Conclusions

In conclusion, the modified posterolateral inverted L-shaped approach has the advantages of small soft tissue dissection, fracture reduction under direct vision, easy internal fixation, and lower risk of neurovascular injury. Therefore, we recommend this approach as an alternative treatment for isolated posterolateral tibial plateau fractures.
